# Reduction in All-Cause Mortality with Fluticasone Furoate/Umeclidinium/Vilanterol in Patients with Chronic Obstructive Pulmonary Disease

**DOI:** 10.1164/rccm.201911-2207OC

**Published:** 2020-06-15

**Authors:** David A. Lipson, Courtney Crim, Gerard J. Criner, Nicola C. Day, Mark T. Dransfield, David M. G. Halpin, MeiLan K. Han, C. Elaine Jones, Sally Kilbride, Peter Lange, David A. Lomas, Sally Lettis, Pamela Manchester, Neil Martin, Dawn Midwinter, Andrea Morris, Steven J. Pascoe, Dave Singh, Robert A. Wise

**Affiliations:** ^1^ Clinical Sciences; ^9^ Development, R&D, and; ^14^ Global Clinical Science and Delivery, GlaxoSmithKline, Collegeville, Pennsylvania; ^2^ Pulmonary, Allergy and Critical Care Division, Department of Medicine, Perelman School of Medicine, University of Pennsylvania, Philadelphia, Pennsylvania; ^3^ Clinical Sciences, GlaxoSmithKline, Research Triangle Park, North Carolina; ^4^ Pulmonary and Critical Care Medicine, Lewis Katz School of Medicine at Temple University, Philadelphia, Pennsylvania; ^5^ Safety and Medical Governance and; ^10^ Biostatistics, GlaxoSmithKline, Uxbridge, Middlesex, United Kingdom; ^6^ Division of Pulmonary, Allergy, and Critical Care Medicine, Lung Health Center, University of Alabama at Birmingham, Birmingham, Alabama; ^7^ University of Exeter Medical School, College of Medicine and Health, University of Exeter, Exeter, United Kingdom; ^8^ University of Michigan, Pulmonary and Critical Care, Ann Arbor, Michigan; ^11^ Section of Epidemiology, Department of Public Health, University of Copenhagen, Copenhagen, Denmark; ^12^ Medical Department, Pulmonary Section, Herlev–Gentofte Hospital, Herlev, Denmark; ^13^ UCL Respiratory, University College London, London, United Kingdom; ^15^ Global Medical Affairs, GlaxoSmithKline, Brentford, Middlesex, United Kingdom; ^16^ Institute for Lung Health, University of Leicester, Leicester, United Kingdom; ^17^ Centre for Respiratory Medicine and Allergy, Institute of Inflammation and Repair, Manchester Academic Health Science Centre, The University of Manchester, Manchester University NHS Foundation Trust, Manchester, United Kingdom; ^18^ Division of Pulmonary and Critical Care Medicine, Johns Hopkins University School of Medicine, Baltimore, Maryland; and; ^19^ New York–Presbyterian Hospital/Weill Cornell Medical Center, New York, New York

**Keywords:** COPD, triple therapy, mortality, survival

## Abstract

**Rationale:** The IMPACT (Informing the Pathway of Chronic Obstructive Pulmonary Disease Treatment) trial demonstrated a significant reduction in all-cause mortality (ACM) risk with fluticasone furoate/umeclidinium/vilanterol (FF/UMEC/VI) versus UMEC/VI in patients with chronic obstructive pulmonary disease (COPD) at risk of future exacerbations. Five hundred seventy-four patients were censored in the original analysis owing to incomplete vital status information.

**Objectives:** Report ACM and impact of stepping down therapy, following collection of additional vital status data.

**Methods:** Patients were randomized 2:2:1 to FF/UMEC/VI 100/62.5/25 μg, FF/VI 100/25 μg, or UMEC/VI 62.5/25 μg following a run-in on their COPD therapies. Time to ACM was prespecified. Additional vital status data collection and subsequent analyses were performed *post hoc*.

**Measurements and Main Results:** We report vital status data for 99.6% of the intention-to-treat population (*n* = 10,355), documenting 98 (2.36%) deaths on FF/UMEC/VI, 109 (2.64%) on FF/VI, and 66 (3.19%) on UMEC/VI. For FF/UMEC/VI, the hazard ratio for death was 0.72 (95% confidence interval, 0.53–0.99; *P* = 0.042) versus UMEC/VI and 0.89 (95% confidence interval, 0.67–1.16; *P* = 0.387) versus FF/VI. Independent adjudication confirmed lower rates of cardiovascular and respiratory death and death associated with the patient’s COPD.

**Conclusions:** In this secondary analysis of an efficacy outcome from the IMPACT trial, once-daily single-inhaler FF/UMEC/VI triple therapy reduced the risk of ACM versus UMEC/VI in patients with symptomatic COPD and a history of exacerbations.

**At a Glance Commentary**

**Scientific Knowledge on the Subject**

Previous studies have suggested that inhaled corticosteroids convey a survival benefit in patients with chronic obstructive pulmonary disease.

**What This Study Adds to the Field**

This study reports a statistically significant reduction in the risk of all-cause mortality comparing fluticasone furoate/umeclidinium/vilanterol (inhaled corticosteroid/long-acting muscarinic antagonist/long-acting β_2_-agonist) with umeclidinium/vilanterol (long-acting muscarinic antagonist/long-acting β_2_-agonist) in the IMPACT (Informing the Pathway of Chronic Obstructive Pulmonary Disease Treatment) trial following additional collection and analysis of vital status data.

No pharmacologic therapy to date has prospectively demonstrated a reduction in all-cause mortality (ACM) in patients with chronic obstructive pulmonary disease (COPD). Only smoking cessation (1), oxygen therapy in severely hypoxemic patients (2, 3), and lung volume reduction surgery in select individuals (4) have been shown to decrease mortality. Previous studies, such as TORCH (5), INSPIRE (6), UPLIFT (7), and SUMMIT (8) suggested a benefit for survival with pharmacologic therapy but either did not achieve statistical significance or were limited by methodologic considerations.

The IMPACT (Informing the Pathway of COPD Treatment) trial (NCT02164513, CTT116855) was a 52-week phase III, randomized, double-blind, parallel-group, multicenter trial that compared the efficacy, safety, and tolerability of once-daily single-inhaler triple therapy containing an inhaled corticosteroid/long-acting muscarinic antagonist/long-acting β_2_-agonist (ICS/LAMA/LABA; fluticasone furoate/umeclidinium/vilanterol [FF/UMEC/VI]) versus ICS/LABA (FF/VI) or LABA/LAMA (UMEC/VI) dual therapy (9). The primary efficacy and safety results have been previously reported (10). The trial demonstrated significant beneficial outcomes for FF/UMEC/VI therapy compared with both dual therapies specifically including a reduction in moderate/severe exacerbations and COPD hospitalizations and improved lung function and health-related quality of life. The safety profile of triple therapy was like that of the known profiles of the individual molecules.

IMPACT also demonstrated a potentially clinically relevant mortality difference including reduction in the risk of on-treatment all-cause mortality and all-cause mortality including off-treatment data in the intent-to-treat (ITT) population, comparing FF/UMEC/VI with UMEC/VI. However, 574 (5.5%) subjects were censored in the original Week 52 analysis that included off-treatment data because of incomplete vital status information, as not all investigators provided vital status data for their subjects at Week 52 after discontinuation of assigned therapy or withdrawal from the study (10). Because of the amount of missing data in the previous results, we felt caution was warranted in the interpretation of the all-cause mortality finding. We now report robust findings of all-cause mortality following collection of additional vital status data at nominal Week 52 representing 99.6% of the study population. In addition, the IMPACT trial design allowed participants to remain on their current COPD therapies prior to randomization, rather than have an artificial “stabilization” or withdrawal of therapy during the run-in period. This was done to mimic therapeutic switch and step down performed in routine clinical practice, thereby improving the generalizability of the trial results. This design affords the opportunity to understand outcomes for participants who entered the trial on differing therapies. We also report on outcomes of patients who entered the trial on inhaled triple therapy and on regimens containing an ICS to understand outcomes of patients who undergo step down or switch in therapy. Some of these data have been previously presented in the form of an abstract (11).

## Methods

### Patient Population

IMPACT randomized 10,355 patients in a 2:2:1 fashion to FF/UMEC/VI 100/62.5/25 μg, FF/VI 100/25 μg, and UMEC/VI 62.5/25 μg, respectively, and were included in the ITT population. Eligible participants had symptomatic COPD with a FEV_1_ <50% of predicted and a history of ≥1 moderate or severe (hospitalized) exacerbation or FEV_1_ of 50% to <80% of predicted and ≥2 moderate or 1 severe exacerbation in the previous year. A current diagnosis of asthma was exclusionary (10).

The total study duration consisted of a 2-week run-in period where participants remained on their own medication, a 52-week treatment period, and a 1-week safety follow-up.

Patients who permanently discontinued study treatment before the end of the 52-week treatment period but agreed to continue in the study were followed by the investigator until the end of the patients’ planned 52-week participation to capture important efficacy and safety assessments, including adverse events, exacerbations, and vital status. Those who discontinued their medications and withdrew from the study were expected to have vital status recorded at the 52-week postrandomization date. All serious adverse reports and deaths within the study were independently adjudicated to determine the primary cause of death.

The study was performed in 37 countries between June 2014 and July 2017 in accordance with Good Clinical Practice and the Declaration of Helsinki. The study received local Institutional Review Board/Independent Ethics Committee approval and all participants provided signed informed consent.

### Definition of All-Cause Mortality

Times to ACM (on-treatment and on/off-treatment) were prespecified “Other” efficacy endpoints in the IMPACT protocol and have previously been reported (10). Here we also include *post hoc* analyses of time to ACM including a near-complete vital status dataset following a challenging global collection of data.

A death was defined as “on-treatment” if the actual date of death occurred up to 7 days after the last day of treatment and considered to be “off-treatment” if the actual date of death occurred more than 7 days after the last day of treatment and up to within 7 days of the projected Week 52 date (Figure E1 in the online supplement).

### Statistical Considerations and Tipping Point Analyses

To control for the type I error in the IMPACT trial, the truncated Hochberg method was used in a closed testing hierarchy across the coprimary and key secondary treatment comparisons. Because all tests within the prespecified statistical hierarchy achieved statistical significance (*P* < 0.001), significance is inferred for all other endpoints and treatment comparisons with a *P* value <0.05 as stated in the IMPACT protocol.

Time to ACM was analyzed using a Cox proportional hazards model with covariates of treatment group, age (at screening), and sex. Kaplan-Meier figures showing probability of patients with an event over time for each treatment group are presented.

To assess the impact of missing vital status data at Week 52 (*n* = 42 of 10,355), tipping point analyses were conducted for the treatment comparison of FF/UMEC/VI compared with UMEC/VI by using multiple imputation for the time to event in participants censored prior to Week 52 using the methods proposed by Jackson and colleagues (12) (Figures E2A and E2B) and by imputing all possible combinations of outcomes for the logistic regression methodology (Figure E2C).

## Results

Participants (*N* = 10,355) with symptomatic COPD and a history of exacerbations were randomized into the ITT population and received study medication. In total, 9,087 (88%) completed the trial with 7,991 (77%) completing the trial on investigational therapy.

Baseline study demographics are shown in Table 1. Most participants (66%) were male, and the mean age was 65.3 years. There were no clinically relevant differences in participant characteristics between the overall treatment groups. However, participants who entered the study on a triple therapy or an ICS-containing regimen had lower lung function and greater history of hospitalization in the previous 12 months and were less likely to be a current smoker compared with those who entered on a dual or monotherapy, or non–ICS-containing regimen (Table 2). The participants who entered on triple therapy or ICS-containing therapy also had greater rates of exacerbation during the study suggesting they carried greater risk (Table 3).

**Table 1.** Baseline Patient Demographics (ITT Population)

	FF/UMEC/VI (*n* = *4,151*)	FF/VI (*n* = *4,134*)	UMEC/VI (*n* = *2,070*)	Overall (*N* = *10,355*)
Age, yr, mean (SD)	65.3 (8.2)	65.3 (8.3)	65.2 (8.3)	65.3 (8.3)
Sex, M, *n* (%)	2,766 (67)	2,748 (66)	1,356 (66)	6,870 (66)
Former smoker, *n* (%)	2,715 (65)	2,711 (66)	1,342 (65)	6,768 (65)
Post-bronchodilator FEV_1_% predicted, mean (SD)	45.7 (15.0)	45.5 (14.8)	45.4 (14.7)	45.5 (14.8)
COPD exacerbations in prior year, *n* (%)				
<2 moderate and no severe	1,198 (29)	1,242 (30)	616 (30)	3,056 (30)
≥2 moderate or ≥1 severe	2,953 (71)	2,892 (70)	1,454 (70)	7,299 (70)
≥2 severe	147 (4)	148 (4)	76 (4)	371 (4)
Baseline COPD medications at screening*, *n* (%)				
ICS + LABA + LAMA	1,672 (40)	1,647 (40)	864 (42)	4,183 (40)
ICS + LABA	1,354 (33)	1,340 (32)	647 (31)	3,341 (32)
LAMA + LABA	389 (9)	349 (8)	196 (9)	934 (9)
LAMA	304 (7)	365 (9)	162 (8)	831 (8)

*Definition of abbreviations*: COPD = chronic obstructive pulmonary disease; FF = fluticasone furoate; ICS = inhaled corticosteroid; ITT = intent-to-treat; LABA = long-acting β_2_-agonist; LAMA = long-acting muscarinic antagonist; UMEC = umeclidinium; VI = vilanterol.

* Medication taken between date of screening −3 days and date of screening (inclusive).

**Table 2.** Baseline Characteristics of Subjects Entering the Trial on a Triple Therapy or an ICS-Containing Regimen

Baseline Characteristic	On Triple Therapy at Screening* (*n* = *4,183*)	No Triple Therapy at Screening* (*n* = *6,172*)	ICS Use at Screening* (*n* = *7,960*)	No ICS Use at Screening* (*n* = *2,395*)
Age, yr, *n*	4,183	6,172	7,960	2,395
Mean (SD)	65.6 (8.1)	65.1 (8.4)	65.2 (8.3)	65.4 (8.2)
Sex, *n*	4,183	6,172	7,960	2,395
Male, *n* (%)	2,733 (65)	4,137 (67)	5,207 (65)	1,663 (69)
Female, *n* (%)	1,450 (35)	2,035 (33)	2,753 (35)	732 (31)
Smoking status, *n*	4,183	6,172	7,960	2,395
Current smoker, *n* (%)	1,294 (31)	2,293 (37)	2,597 (33)	990 (41)
Former smoker, *n* (%)	2,889 (69)	3,879 (63)	5,363 (67)	1,405 (59)
Post-bronchodilator % predicted FEV_1_, *n*	4,182	6,165	7,955	2,392
Mean (SD)	42.9 (14.1)	47.4 (15.1)	44.8 (14.7)	47.9 (14.9)
GOLD grade, *n*	4,182	6,165	7,955	2,392
GOLD 1, *n* (%)	3 (<1)	19 (<1)	12 (<1)	10 (<1)
GOLD 2, *n* (%)	1,206 (29)	2,513 (41)	2,729 (34)	990 (41)
GOLD 3, *n* (%)	2,173 (52)	2,809 (46)	3,886 (49)	1,096 (46)
GOLD 4, *n* (%)	800 (19)	824 (13)	1,328 (17)	296 (12)
Exacerbation history, *n*	4,183	6,172	7,960	2,395
<2 Moderate and no severe exacerbations in the past year, *n* (%)	1,258 (30)	1,798 (29)	2,301 (29)	755 (32)
≥2 Moderate or ≥1 severe exacerbation in the past year, *n* (%)	2,925 (70)	4,374 (71)	5,659 (71)	1,640 (68)
≥1 Severe exacerbation, *n* (%)	1,274 (30)	1,397 (23)	2,120 (27)	551 (23)

*Definition of abbreviations*: GOLD = Global Initiative for Chronic Obstructive Lung Disease; ICS = inhaled corticosteroid.

* Medication taken between date of screening −3 days and date of screening (inclusive).

**Table 3.** Rates of On-Treatment Moderate/Severe Exacerbations in IMPACT by Medication at Study Entry

Baseline Medication*	FF/UMEC/VI (95% CI)	FF/VI (95% CI)	UMEC/VI (95% CI)
Overall	0.91 (0.87–0.95)	1.07 (1.02–1.12)	1.21 (1.14–1.29)
ICS/LAMA/LABA	1.21 (1.13–1.28)	1.43 (1.35–1.53)	1.72 (1.58–1.87)
ICS/LABA	0.70 (0.64–0.77)	0.85 (0.78–0.92)	0.94 (0.83–1.06)
LAMA/LABA	0.84 (0.73–0.98)	1.11 (0.95–1.29)	1.05 (0.86–1.29)
LAMA	0.65 (0.54–0.78)	0.75 (0.64–0.89)	0.61 (0.47–0.80)

*Definition of abbreviations*: CI = confidence interval; FF = fluticasone furoate; ICS = inhaled corticosteroid; IMPACT = Informing the Pathway of Chronic Obstructive Pulmonary Disease Treatment; LABA = long-acting β_2_-agonist; LAMA = long-acting muscarinic antagonist; UMEC = umeclidinium; VI = vilanterol.

Medication classes are mutually exclusive.

* Medication taken between date of screening −3 days and date of screening (inclusive).

### On-Treatment All-Cause Mortality

**Figure 1.**
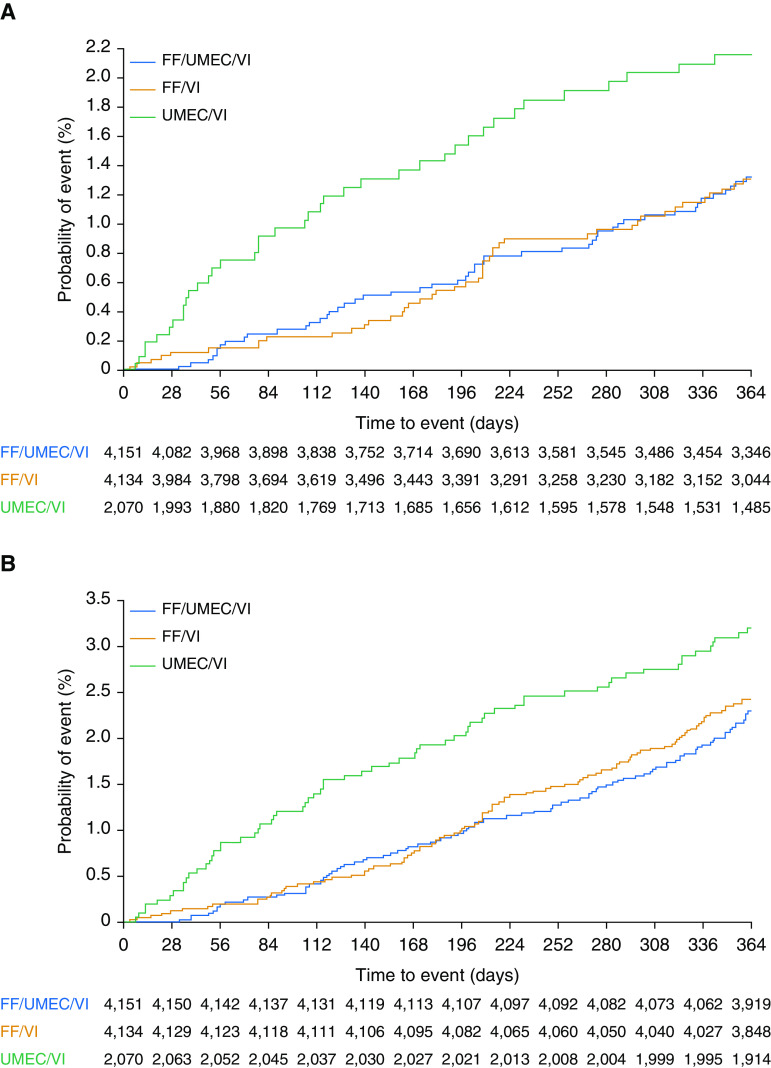
Kaplan-Meier plots of time to all-cause mortality for (*A*) on-treatment deaths and (*B*) on/off-treatment deaths. FF = fluticasone furoate; UMEC = umeclidinium; VI = vilanterol.

As originally reported (10), there were 50 (1.20%) on-treatment deaths in the FF/UMEC/VI arm (*n* = 4,151), 49 (1.19%) in the FF/VI arm (*n* = 4,134), and 39 (1.88%) in the UMEC/VI arm (*n* = 2,070). The hazard ratio (HR) for on-treatment ACM was 0.58 (95% confidence interval [CI], 0.38–0.88; *P* = 0.011) for the comparison of FF/UMEC/VI with UMEC/VI and 0.61 (95% CI, 0.40–0.93; *P* = 0.022) for the comparison of FF/VI with UMEC/VI (Figure 1A). Additional data collection that gathered off-treatment vital status information does not impact the prespecified on-treatment all-cause mortality analyses and findings.

Independent adjudication of the primary cause of death confirmed lower rates of cardiovascular death, respiratory death, and death associated with the patient’s underlying COPD when on either randomized ICS-containing arm compared with UMEC/VI. Similar findings were observed when off-treatment adjudicated data were included. (Table E1).

### On/Off-Treatment All-Cause Mortality Including Additional Vital Status Data

Additional *post hoc* data collection now provides vital status at Week 52 for 99.6% of the ITT population with 42 subjects censored in the analyses because of missing data. Twenty-seven additional off-treatment deaths were identified in the *post hoc* collection of vital status information (9 on FF/UMEC/VI, 12 on FF/VI, and 6 on UMEC/VI). In total, there were 98 (2.36%) deaths on FF/UMEC/VI, 109 (2.64%) on FF/VI, and 66 (3.19%) on UMEC/VI. Time to ACM, including off-treatment data with the additional vital status collection, demonstrated an HR for ACM of 0.72 for patients treated with FF/UMEC/VI compared with UMEC/VI (95% CI, 0.53–0.99, *P* = 0.042). The HR for FF/VI versus UMEC/VI was 0.82 (95% CI, 0.60–1.11, *P* = 0.190) (Figure 1B).

**Figure 2.**
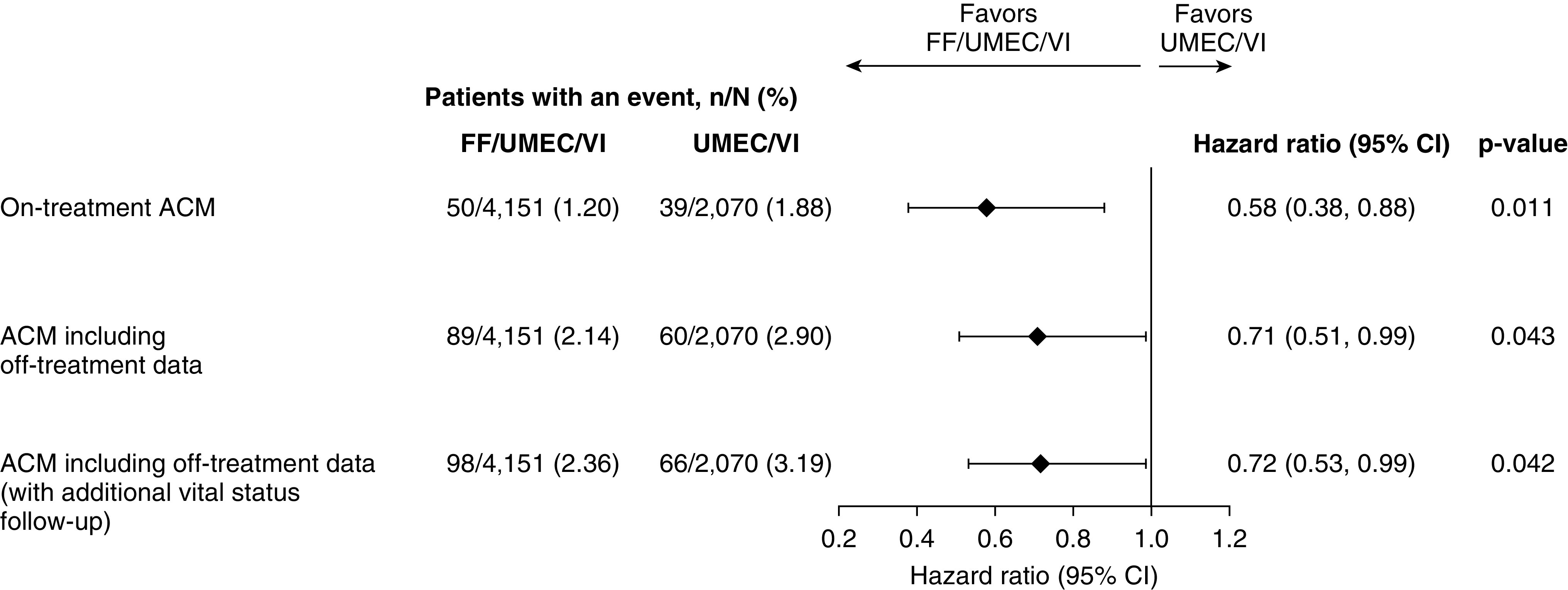
Forest plot of ACM analyses and hazard ratios FF/UMEC/VI versus UMEC/VI. ACM = all-cause mortality; CI = confidence interval; FF = fluticasone furoate; UMEC = umeclidinium; VI = vilanterol.

Figure 2 illustrates the results of the various presented analyses including the originally reported findings.

Tipping point analyses conducted for the treatment comparison of FF/UMEC/VI compared with UMEC/VI using multiple imputation for the time to event in participants censored prior to Week 52 demonstrated that if all patients on UMEC/VI with censored data are imputed as alive at the end of 52 weeks the postwithdrawal hazard for FF/UMEC/VI would need to be approximately 10 times higher than the prewithdrawal hazard before losing statistical significance. Similarly, if the patients on UMEC/VI with censored data are assumed to have a postwithdrawal hazard the same as the prewithdrawal hazard (i.e., it is assumed that the missing data for UMEC/VI are missing at random), then the postwithdrawal hazard for FF/UMEC/VI would need to be approximately 14 times higher than the prewithdrawal hazard before losing statistical significance. These extreme assumptions are unlikely and support the conclusion that the observed survival finding is robust to missing data (Figure E2).

### Effect of Step Down from Triple and Switch of Therapy

Table E2 summarizes ACM by the patients’ COPD medication at study entry. Forty percent of participants (*n* = 4,183) entered the study on a triple therapy (ICS + LAMA + LABA) regimen. Because of the randomization scheme, approximately 40% of these patients were maintained on triple therapy, 40% were stepped down to ICS/LABA (removal of the LAMA component), and 20% were stepped down to a LAMA/LABA (removal of the ICS component).

**Figure 3.**
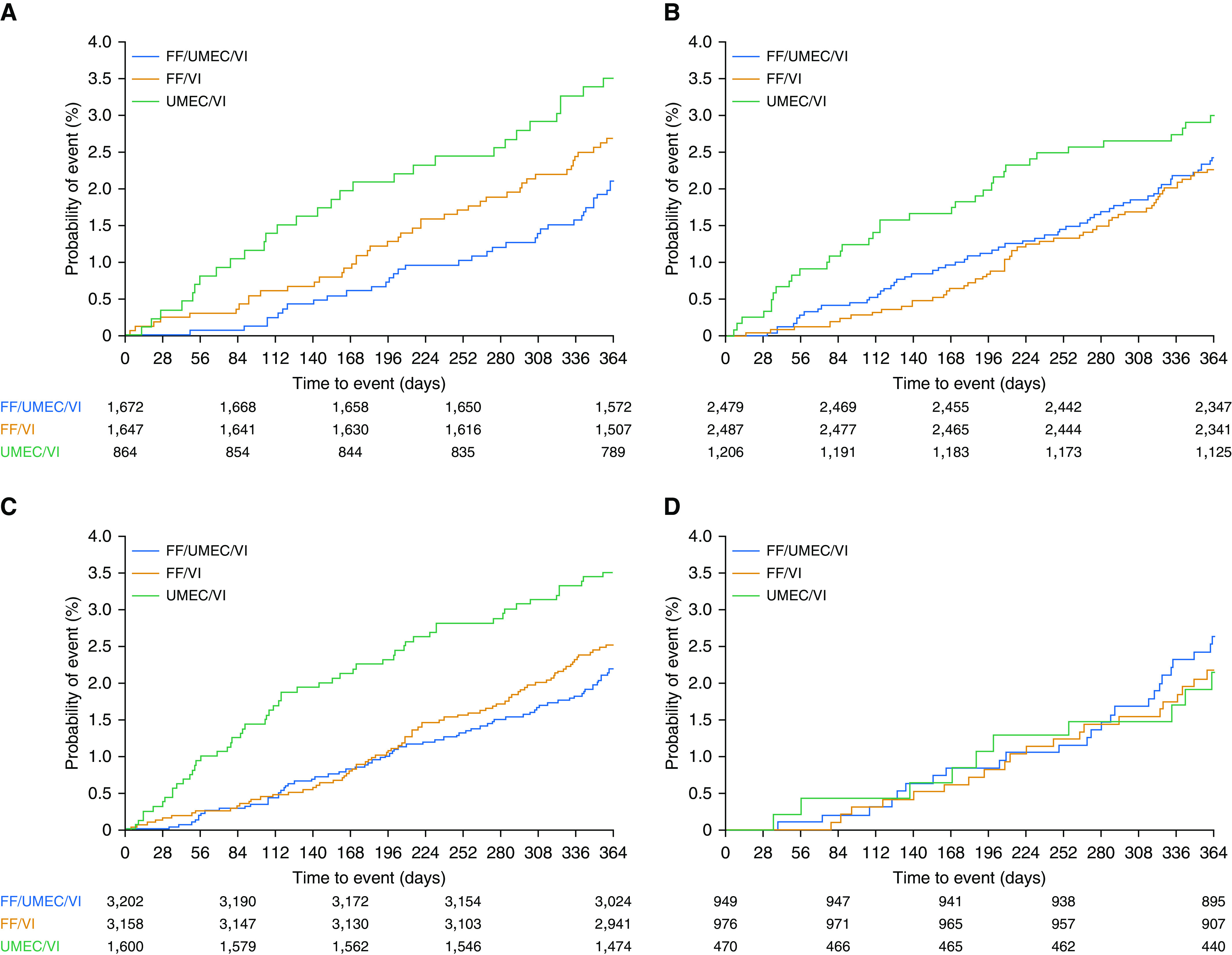

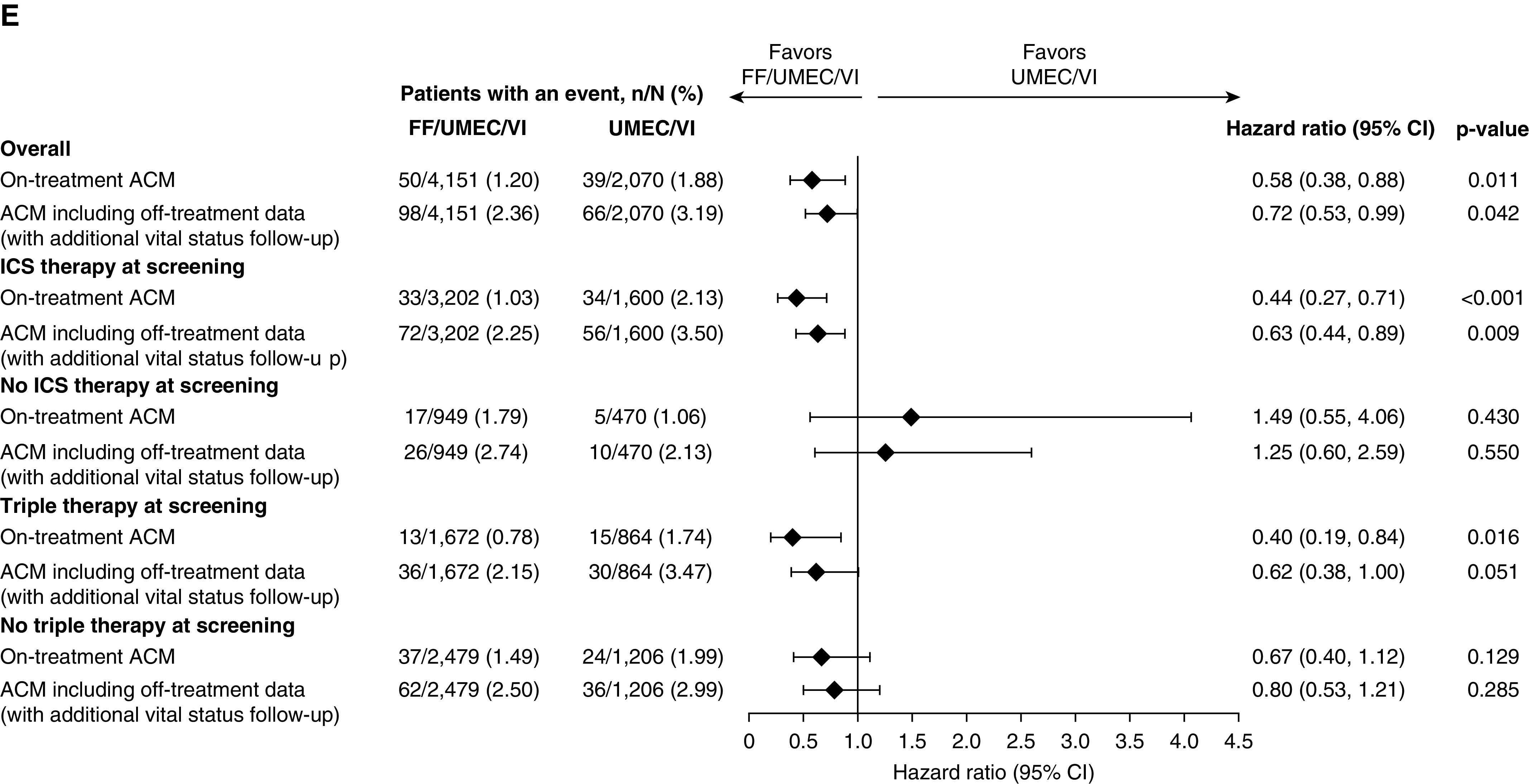
ACM by triple therapy or ICS use at screening*: (*A*) triple therapy at screening, (*B*) no triple therapy at screening, (*C*) ICS use at screening, (*D*) no ICS use at screening, and (*E*) forest plot of ACM analysis by therapy at screening. (*A*–*D*) Kaplan-Meier plots of ACM including off-treatment data (with additional vital status follow-up). ACM = all-cause mortality; CI = confidence interval; FF = fluticasone furoate; ICS = inhaled corticosteroid; UMEC = umeclidinium; VI = vilanterol. *Medication taken between date of screening −3 days and date of screening (inclusive).

In the analysis including the additional follow-up data in participants who entered the study on a triple therapy, the study suggested a reduced risk of on/off-treatment death for patients maintained on a triple therapy compared with those who underwent step down to either dual therapy (Figure 3A) with an HR of 0.71 (95% CI, 0.46–1.10; *P* = 0.124) compared with patients stepped down to ICS/LABA, and an HR of 0.62 (95% CI, 0.38–1.00; *P* = 0.051) compared with patients stepped down to LAMA/LABA, although these reductions did not achieve statistical significance.

In those patients who entered the study on medications other than a triple regimen, risk of all-cause mortality was numerically lower for patients who were randomized to either triple therapy or to ICS/LABA compared with those randomized to LAMA/LABA (Figure 3B). For participants in this subgroup randomized to triple therapy the HR for risk of ACM was 1.03 (95% CI, 0.72–1.47; *P* = 0.870) compared with ICS/LABA and 0.80 (95% CI, 0.53–1.21; *P* = 0.285) compared with LAMA/LABA (Figure 3E).

### Effect of Previous ICS Use on All-Cause Mortality

Most participants (76.9%, *n* = 7,960) who entered the study were on a medication regimen that contained an ICS. In these patients, mortality was lower if they were maintained on an ICS-containing regimen compared with LAMA/LABA (Figure 3C). The HR for risk of ACM in this subgroup was 0.82 (95% CI, 0.60–1.13; *P* = 0.229) for patients randomized to triple therapy compared with ICS/LABA and 0.63 (95% CI, 0.44–0.89; *P* = 0.009) compared with LAMA/LABA (Figure 3E). There was no apparent difference in mortality in the smaller number of participants (23.1%, *n* = 2,395) who entered the study on a non–ICS-containing medication regimen (Figure 3D), although interpretation is limited given the relatively smaller size of this group and lower number of deaths.

### Analysis of All-Cause Mortality by Time Intervals after Randomization

Analysis of ACM by time interval (death within 30, 60, or 180 d after randomization) demonstrated a statistically significant benefit for FF/UMEC/VI compared with UMEC/VI within 60 and 180 days after randomization. There were no deaths in the FF/UMEC/VI arm, 5 in the FF/VI arm, and 7 in the UMEC/VI arm within 30 days of randomization; no analysis was performed owing to the small number of events (Figures E3 and E4).

## Discussion

We demonstrate that treatment of symptomatic patients with COPD and a history of exacerbation with FF/UMEC/VI significantly reduced the risk of all-cause mortality compared with the dual bronchodilator UMEC/VI. This finding was consistently observed in the on-treatment analyses, the analyses that included off-treatment data, and in sensitivity tipping point analyses. These data extend previous studies that suggested a reduction in mortality using ICS-containing medications in patients with COPD (5, 6, 8, 13, 14).

We confirm a survival benefit to ICS-containing therapy in a predefined, prospective analysis. The survival benefit has been previously suggested in other studies, but either did not reach predetermined levels of statistical significance or were performed *post hoc*. The TORCH study (5) demonstrated a 17.5% (95% CI, −0.2 to 31.9) reduction in the hazard of death in the combination group using salmeterol/fluticasone propionate compared with placebo (*P* = 0.052) and only missed statistical significance owing to an interim analysis that raised the threshold for significance. The INSPIRE study (6) demonstrated a 52% reduction in the hazard of on-treatment ACM with salmeterol/fluticasone propionate compared with tiotropium (HR, 0.48 [95% CI, 0.27 to 0.85]; *P* = 0.012), although this analysis was performed *post hoc* on a safety endpoint with incomplete follow-up. The SUMMIT study (8) demonstrated a 12.2% reduction in the hazard of ACM for FF/VI compared with placebo (HR, 0.88 [95% CI, 0.74 to 1.04]; *P* = 0.137) in a milder COPD population with either the presence or high risk of cardiovascular disease. The endpoint did not reach statistical significance in the study, although that may be because that study was powered assuming a 30% reduction in mortality in a much milder patient population. A *post hoc*, stratified, safety-pooled analysis of fatal adverse events of ICS-containing therapy in three 52-week studies suggested a 29% reduction in mortality, although this did not reach statistical significance (HR, 0.71 [95% CI, 0.50–1.02]; *P* = 0.066) (13). Interestingly, a recent Bayesian network meta-analysis of 219 trials found that both ICS/LAMA/LABA and ICS/LABA were associated with a statistically significantly higher probability of reducing mortality compared with placebo (odds ratio [OR], 0.74 [95% credible interval, 0.59 to 0.93]; posterior probability of OR > 1 [*P*(OR > 1)] = 0.004; and OR, 0.86 [95% credible interval, 0.76 to 0.98]; *P*[OR > 1] = 0.015; respectively). Thus, to put these data into perspective, IMPACT has now prospectively confirmed a survival benefit with ICS-containing therapy that had previously been suggested in patients with COPD.

It is likely that the finding of reduction in the risk of all-cause mortality was confirmed in IMPACT because of the clinical severity of the population in the study and the significant efficacy observed with the addition of the ICS FF in a highly symptomatic group of patients with frequent moderate or severe exacerbations. In IMPACT we observed a 25% reduction in the rate of on-treatment moderate and severe COPD exacerbations comparing FF/UMEC/VI with UMEC/VI as well as a 34% reduction in COPD hospitalizations for this comparison (10). The reduction in recurrent exacerbation events likely led to improved patient well-being and reduced hospitalization. Reduction in hospitalization likely reduced the known morbidity and mortality associated with hospitalization in patients with COPD (15–18). This is supported by the independently adjudicated findings of reduced cardiovascular death, respiratory death, and death associated with the patient’s underlying COPD compared with an efficacious active comparator.

Our time interval data refute the premise that the difference in all-cause mortality was due to acute ICS withdrawal, as evidenced by the continued reduction in mortality throughout the trial, not only in the first 30 days when the effect of acute ICS withdrawal would be expected to be greatest. These data suggest acute step down of medication did not drive the overall findings; rather, they demonstrate the overall benefit of ICS for this population. The observation of the statistically significant and clinically relevant reduction of on-treatment mortality with FF/VI compared with UMEC/VI further supports that the ICS component drives the survival benefit. The findings of triple compared with FF/VI, and the triple step-down data, demonstrate an additional contribution of the LAMA component to survival when using triple-inhaled therapy in this population.

The survival benefit was observed in participants who entered the trial on ICS. This is not unexpected as these patients appeared at greater risk with lower lung function and higher rates of previous hospitalization at study entry, despite being on ICS. As this population is sicker, as evidenced by higher rates of exacerbations during the trial, and most likely to be hospitalized, one might expect that this is the population that would derive the greatest benefit in the study. Only a minority of patients entered the study not taking an ICS, so we are less able to determine if there is a survival benefit in this smaller subgroup with lower risk at study entry.

Differential response based on prior treatment has been observed in other trials. For example, *post hoc* analyses of patients who were previously treated with ICS in the SUMMIT trial demonstrated a beneficial effect on mortality differing from those who had not been previously treated with ICS (19). Additionally, both LABA and ICS use predicted a higher rate of healthcare-utilized exacerbation in the TIOSPIR study (20). Perhaps this should be expected as patients were on their previous medications for a reason and would likely have different clinical characteristics that would have prompted their physician to use these medications in the first place (19). This also suggests that there is something inherently different about these patients, rather than an effect of withdrawal or switch of medication in IMPACT and in these trials.

The data from patients who entered the trial on an “open” triple of ICS + LAMA + LABA suggest that maintenance on a triple therapy is associated with a trend toward lower risk of death than step down to either dual therapy in a symptomatic patient population at risk for further exacerbation. This supports the benefit of both ICS and LAMA in this population and is of importance as international treatment guidelines suggest consideration of step down of therapy in stable patients (21). However, our findings suggest that physicians should use caution when considering step down in therapy in patients with characteristics that mirror those enrolled in IMPACT.

A limitation of the study was that it was only of 52 weeks in duration. Previous mortality studies have been a minimum of 2–3 years in length to ensure enough events to demonstrate a mortality difference. However, we were able to demonstrate a difference despite being only 1 year in length, likely owing to the high-risk nature of this population. Mortality studies of longer length have shown that the mortality curves between arms generally continue to widen over time, although this is dependent on disease stage and the mode of action of a drug (5, 8). The mortality finding in IMPACT would not be expected to be different from previous longer studies as there is no suggestion from the data or the study population that the benefit would wane over time. Strengths of the study include the large sample size of well-characterized participants with substantial follow-up information. An additional strength is that we evaluated both on-treatment mortality (vital status of subjects while taking assigned therapy) and the mortality of subjects including off-treatment data (including vital status of subjects even after discontinuation of assigned therapy, as ITT). On-treatment data are clinically relevant because they demonstrate expected outcomes related to use of a chronic therapy while the subject is taking the medication; ACM including off-treatment data is important for understanding treatment policy and the impact of differential dropout.

In summary, we have now prospectively confirmed for the first time a reduction in the risk of death using pharmacologic therapy with once-daily inhaled FF/UMEC/VI in symptomatic patients at risk for future exacerbations. We believe that these data are important to healthcare providers and to patients with COPD.

## Supplementary Material

Supplements

Author disclosures
