# Supplementary material for: Reduction in All-Cause Mortality with Fluticasone Furoate/Umeclidinium/Vilanterol in Patients with Chronic Obstructive Pulmonary Disease
Source: Am J Respir Crit Care Med. 2020 Jun 15;201(12):1508–16. doi: 10.1164/rccm.201911-2207OC (PMC7301738; doi:10.1164/rccm.201911-2207OC)
Supplement: Supplements [file rccm.201911-2207OC.html]

Reduction in All-Cause Mortality with Fluticasone Furoate/Umeclidinium/Vilanterol in Patients with Chronic Obstructive Pulmonary Disease | American Journal of Respiratory and Critical Care Medicine

- disclosures.pdf (501 KB)
- lipson\_data\_supplement.pdf (1 MB)
