# Supplementary material for: Reduction in All-Cause Mortality with Fluticasone Furoate/Umeclidinium/Vilanterol in Patients with Chronic Obstructive Pulmonary Disease
Source: Am J Respir Crit Care Med. 2020 Jun 15;201(12):1508–16. doi: 10.1164/rccm.201911-2207OC (PMC7301738; doi:10.1164/rccm.201911-2207OC)
Supplement: Supplements [file rccm.201911-2207OC_lipson_data_supplement.pdf]

Reduction in all-cause mortality with fluticasone furoate/umeclidinium/vilanterol in COPD patients

David A. Lipson MD, Courtney Crim MD, Gerard J. Criner MD, Nicola C. Day PhD, Mark T. Dransfield MD, David M.G. Halpin MD, MeiLan K. Han MD, C. Elaine Jones PhD, Sally Kilbride MSc, Peter Lange MD, David A. Lomas MD PhD, Sally Lettis PhD, Pamela Manchester BS, Neil Martin, MD, Dawn Midwinter MSc, Andrea Morris BS, Steven J. Pascoe MD, Dave Singh MD, Robert A. Wise MD, Fernando J. Martinez MD; on behalf of the IMPACT investigators

Online Data Supplement

Supplement Table E1. Summary of adjudicated deaths including off-treatment data

	FF/UMEC/VI (N=4151)		FF/VI (N=4134)		UMEC/VI (N=2070)	
	**n (%)**	**Rate [#]**	**n (%)**	**Rate [#]**	**n (%)**	**Rate [#]**
Total duration at risk (subject-years)	4088.3		4030.1		1999.3	
**Primary cause of death**						
Total	88 (2)	21.5 [88]	92 (2)	22.8 [92]	58 (3)	29.0 [58]
Cardiovascular	26 (<1)	6.4 [26]	31 (<1)	7.7 [31]	20 (<1)	10.0 [20]
Respiratory	25 (<1)	6.1 [25]	26 (<1)	6.5 [26]	18 (<1)	9.0 [18]
Cancer	13 (<1)	3.2 [13]	7 (<1)	1.7 [7]	6 (<1)	3.0 [6]
Unknown	14 (<1)	3.4 [14]	14 (<1)	3.5 [14]	11 (<1)	5.5 [11]
Other	10 (<1)	2.4 [10]	14 (<1)	3.5 [14]	3 (<1)	1.5 [3]
**Death associated with COPD**						
Yes	34 (<1)	8.3 [34]	36 (<1)	8.9 [36]	25 (1)	12.5 [25]
No	40 (<1)	9.8 [40]	37 (<1)	9.2 [37]	16 (<1)	8.0 [16]
Inadequate information	9 (<1)	2.2 [9]	14 (<1)	3.5 [14]	12 (<1)	6.0 [12]
Indeterminate	5 (<1)	1.2 [5]	5 (<1)	1.2 [5]	5 (<1)	2.5 [5]

COPD, chronic obstructive pulmonary disease; FF, fluticasone furoate; UMEC, umeclidinium; VI, vilanterol. Note: n = Number of subjects, # = Number of events, Rate

is event rate per 1000 subject-years, calculated as the number of events x 1000, divided by the total duration at risk. Previously missing data were not re-adjudicated.

Supplement Table E2. Summary of ACM including off-treatment data by COPD medication at study entry*

COPD medication	FF/UMEC/VI (N=4151)		FF/VI (N=4134)		UMEC/VI (N=2070)	
	**n/N (%)**	**Rate [#]**	**n/N (%)**	**Rate [#]**	**n/N (%)**	**Rate [#]**
ICS + LABA + LAMA	36/1672 (2.15)	0.0214	49/1647 (2.98)	0.0297	30/864 (3.47)	0.0351
ICS + LABA without LAMA	29/1354 (2.14)	0.0213	30/1340 (2.24)	0.0222	22/647 (3.40)	0.0343
LAMA + LABA without ICS	12/389 (3.08)	0.0307	10/349 (2.87)	0.0288	3/196 (1.53)	0.0153
ICS + LAMA without LABA	1/47 (2.13)	0.0211	0/40		0/20	
LAMA without LABA or ICS	6/304 (1.97)	0.0195	6/365 (1.64)	0.0164	3/162 (1.85)	0.0185
ICS without LABA or LAMA	6/129 (4.65)	0.0462	6/131 (4.58)	0.0459	4/69 (5.8)	0.0592
LABA without ICS or LAMA	3/118 (2.54)	0.0251	1/119 (0.84)	0.0083	4/54 (7.41)	0.0755
No ICS, LABA or LAMA	5/138 (3.62)	0.0363	7/143 (4.90)	0.0490	0/58	

ACM, all-cause mortality; FF, fluticasone furoate; ICS, inhaled corticosteroid; LABA, long-acting β_2-agonist; LAMA, long-acting muscarinic antagonist; UMEC, umeclidinium; VI, vilanterol. Note: Data in the table include additional vital status follow-up.

*Medication taken between date of screening -3 days and date of screening (inclusive).

n: number of subjects with event; N: number of subjects in subgroup. Rate is event rate per subject-year, calculated as the number of events divided by the total duration at risk.

Supplement Figures

Supplement Figure E1. Definitions of on- and off-treatment deaths

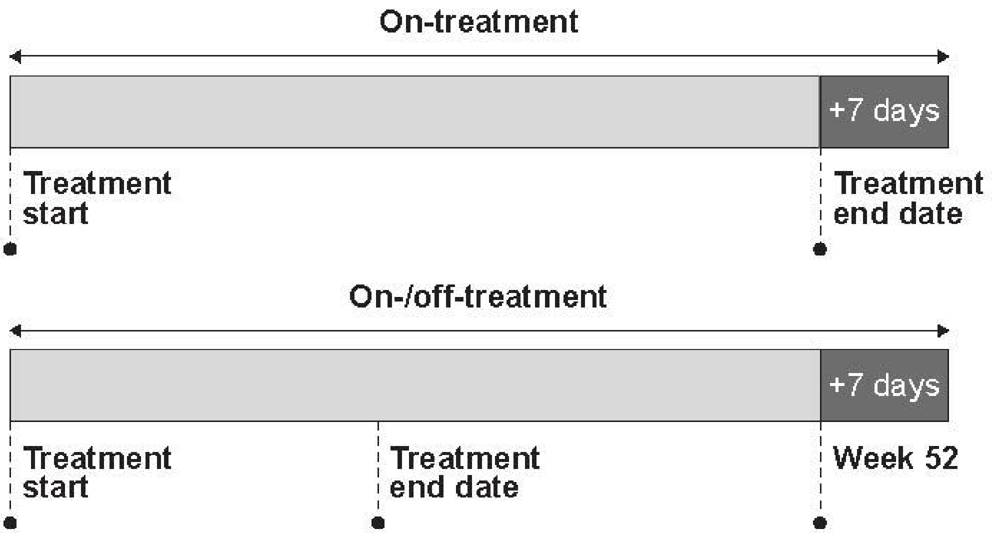

Supplement Figure E2. Tipping point analyses of ACM including off-treatment data for FF/UMEC/VI versus UMEC/VI. A: Analysis with imputation P-value for hazard ratio; B: Analysis with imputation hazard ratio and P-value; C: Analysis with imputation odds ratio and P-value

A

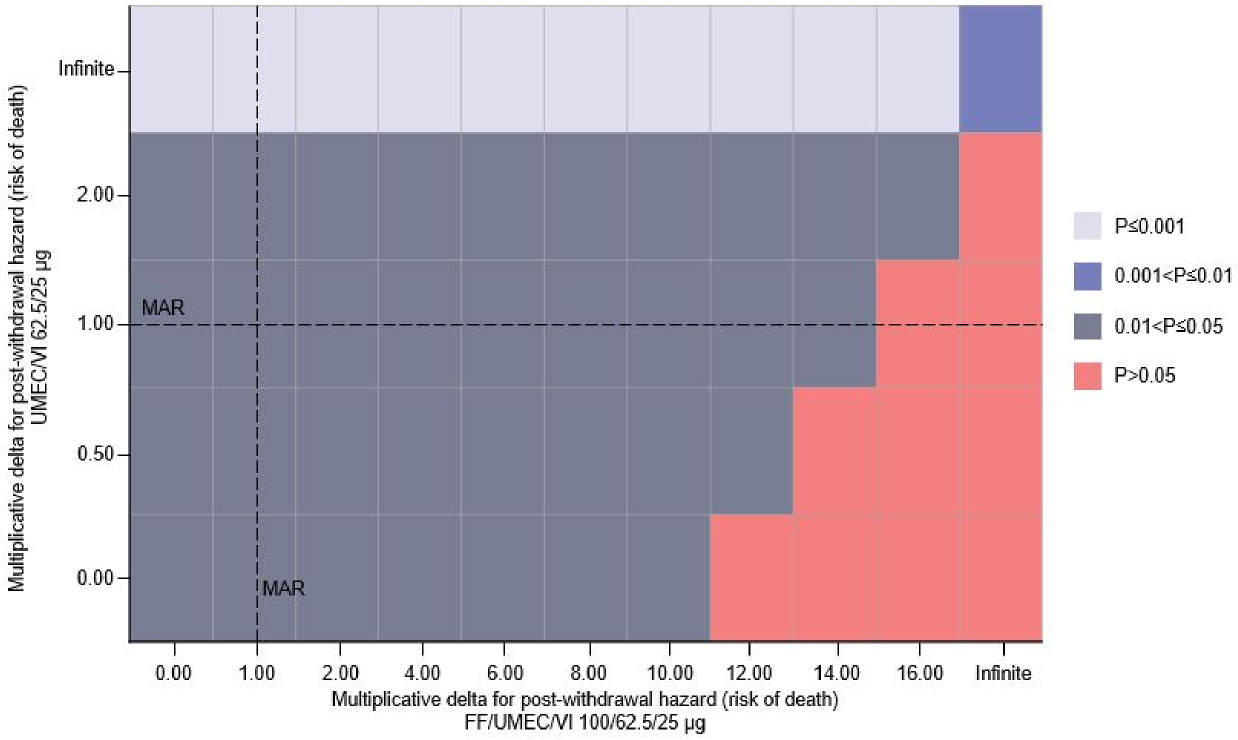

B

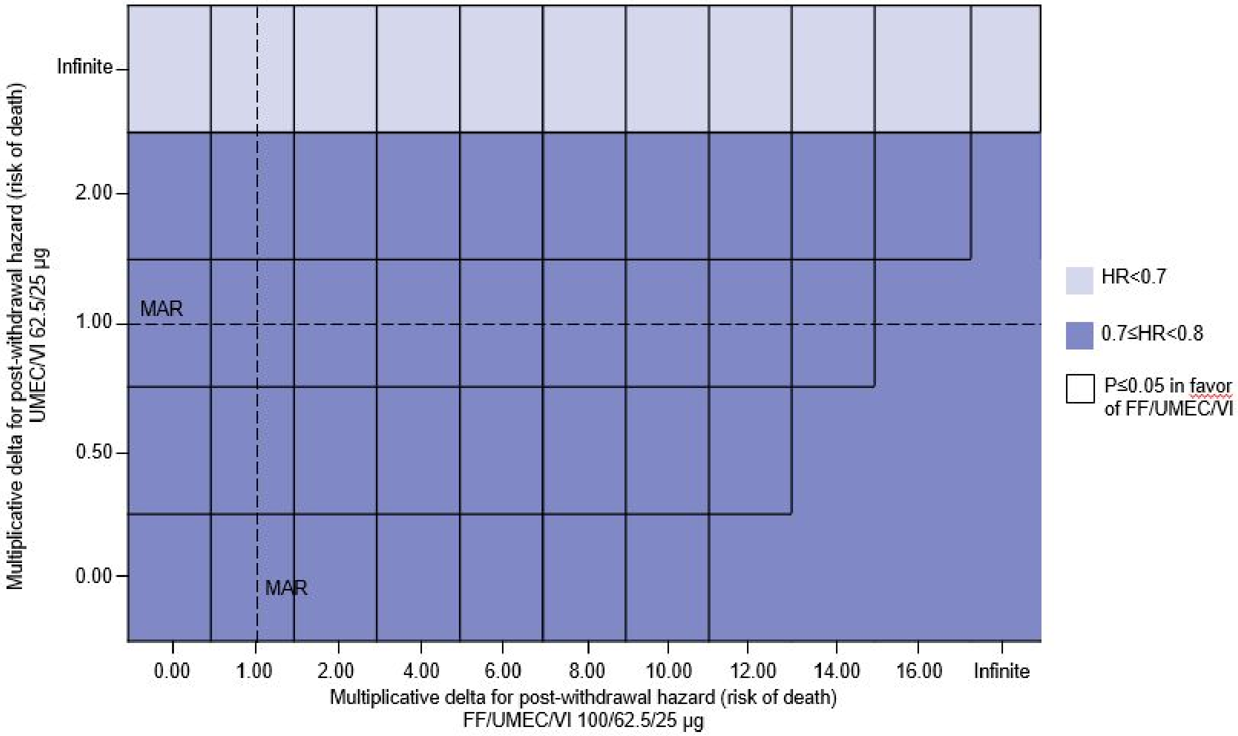

Infinite
2.00
1.00
0.50
0.00
MAR
MAR
Multiplicative delta for post-withdrawal hazard (risk of death)
UMEC/VI 62.5/25 µg
0.00
1.00
2.00
4.00
6.00
8.00
10.00
12.00
14.00
16.00
Infinite
Multiplicative delta for post-withdrawal hazard (risk of death)
FF/UMEC/VI 100/62.5/25 µg
HR<0.7
0.7≤HR<0.8
P≤0.05 in favor of FF/UMEC/VI

C

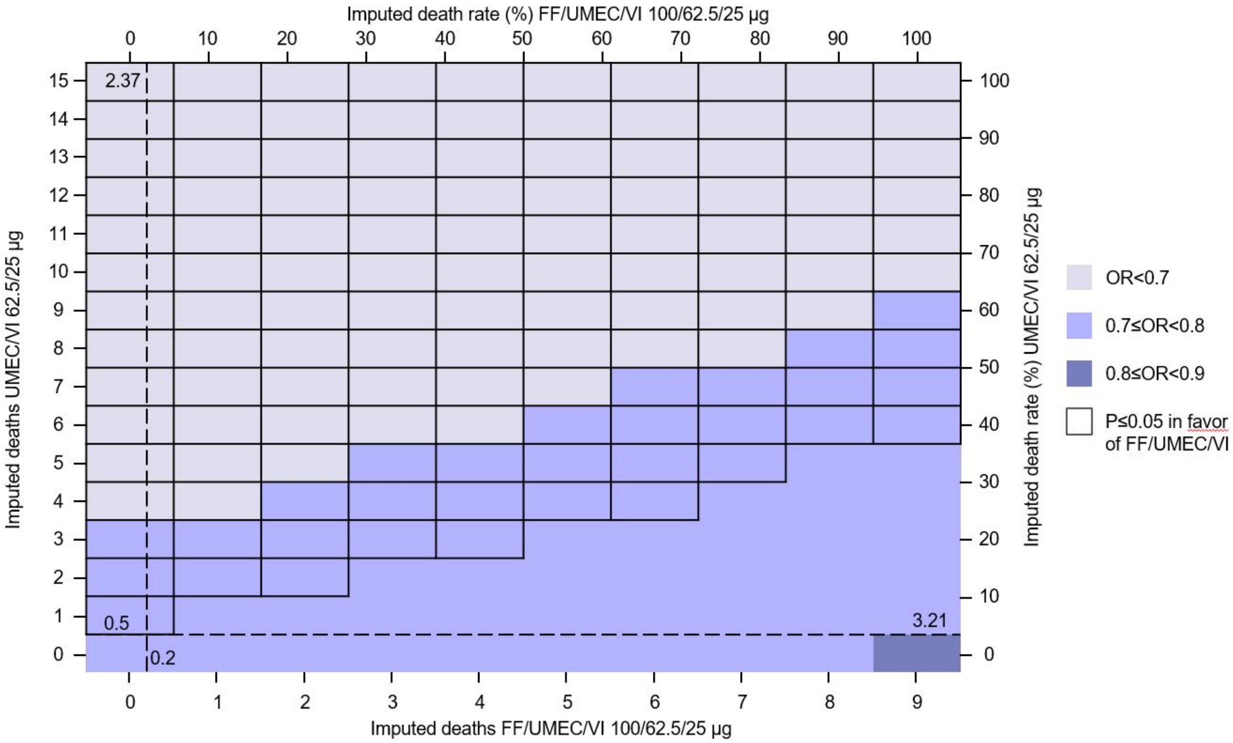

A. If all patients on UMEC/VI with censored data are imputed as alive at the end of 52 weeks the post-withdrawal hazard for FF/UMEC/VI would need to be approximately 10 times higher than the pre-withdrawal hazard before losing statistical significance. If the patients on UMEC/VI with censored data are assumed to have a post-withdrawal hazard the same as the pre-withdrawal hazard (i.e., it is assumed that the missing data for UMEC/VI is missing at random) then the post-withdrawal hazard for FF/UMEC/VI would need to be approximately 14 times higher than the pre-withdrawal hazard before losing statistical significance. **B.** Regardless of the assumption made about the post-withdrawal hazard for either treatment arm, there is at least a 20% reduction in the risk of death (HR<0.80) on FF/UMEC/VI compared with UMEC/VI. **C.** Regardless of the number of patients with missing survival status that we impute as dead or alive on either treatment, the OR is always <0.81 (i.e. the odds of dying is at least 19% lower on

FF/UMEC/VI compared with UMEC/VI [OR=0.803 for all patients on UMEC/VI with censored data imputed as alive and all patients on FF/UMEC/VI with censored data imputed as dead]).

A and **B.** Reference line denotes expected number of deaths under a MAR assumption, i.e. that the hazard for the imputed period is the same as the hazard seen in the observed data. **C.** Reference line denotes expected number of deaths under a missing at random assumption, i.e. that deaths occur at the same rate for patients with a missing survival status at Day 356 as those with a known survival status.

FF, fluticasone furoate; HR, hazard ratio; MAR, missing at random; OR, odds ratio; UMEC, umeclidinium; VI, vilanterol.

Supplement Figure E3. Forest plot of ACM by time interval for FF/UMEC/VI versus UMEC/VI

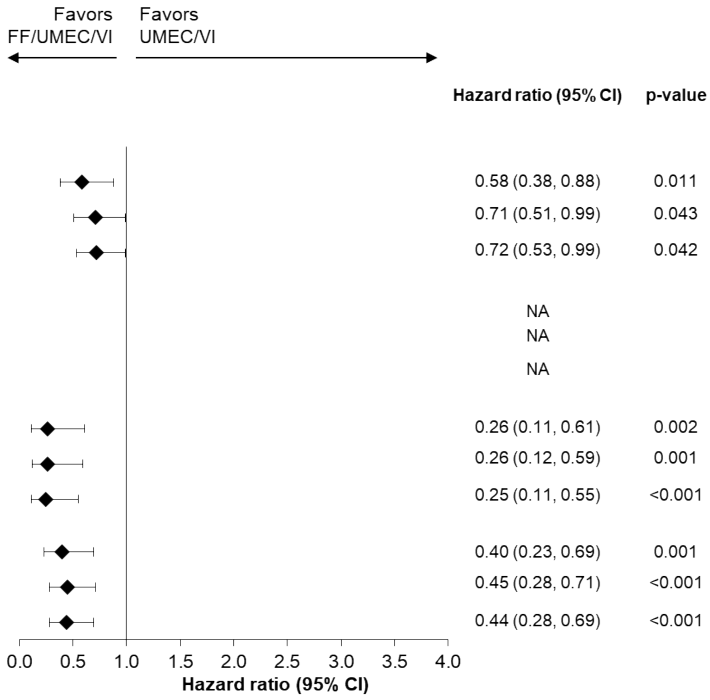

	Patients with an event, n/N (%) FF/UMEC/VI	UMEC/VI	Hazard ratio (95% CI)	p-value
**Overall**				
On-treatment ACM	50/4151 (1.20)	39/2070 (1.88)	0.58 (0.38, 0.88)	0.011
ACM including off-treatment data	89/4151 (2.14)	60/2070 (2.90)	0.71 (0.51, 0.99)	0.043
ACM including off-treatment data (With additional vital status follow-up)	98/4151 (2.36)	66/2070 (3.19)	0.72 (0.53, 0.99)	0.042
**Within 30 days**				
On-treatment ACM	0/4151 (0)	7/2070 (0.34)	NA	
ACM including off-treatment data	0/4151 (0)	7/2070 (0.34)	NA	
ACM including off-treatment data (With additional vital status follow-up)	0/41551 (0)	7/2070 (0.34)	NA	
**Within 60 days**				
On-treatment ACM	8/4151 (0.19)	15/2070 (0.72)	0.26 (0.11, 0.61)	0.002
ACM including off-treatment data	9/4151 (0.22)	17/2070 (0.82)	0.26 (0.12, 0.59)	0.001
ACM including off-treatment data (With additional vital status follow-up)	9/4151 (0.22)	18/2070 (0.87)	0.25 (0.11, 0.55)	<0.001
**Within 180 days**				
On-treatment ACM	23/4151 (0.55)	27/2070 (1.30)	0.40 (0.23, 0.69)	0.001
ACM including off-treatment data	35/4151 (0.84)	38/2070 (1.84)	0.45 (0.28, 0.71)	<0.001
ACM including off-treatment data (With additional vital status follow-up)	36/4151 (0.87)	40/2070 (1.93)	0.44 (0.28, 0.69)	<0.001

ACM, all-cause mortality; CI, confidence interval; FF, fluticasone furoate; UMEC, umeclidinium; VI, vilanterol. Note: Analysis was not performed if there were zero events in one or more of the three treatment arms (FF/UMEC/VI, FF/VI or UMEC/VI).

Supplement Figure E4. Forest plot of ACM by time interval for FF/UMEC/VI versus FF/VI

	Patients with an event, n/N (%) FF/UMEC/VI	FF/VI	Hazard ratio (95% CI)	p-value
**Overall**				
On-treatment ACM	50/4151 (1.20)	49/4134 (1.19)	0.95 (0.64, 1.40)	0.780
ACM including off-treatment data	89/4151 (2.14)	97/4134 (2.35)	0.90 (0.67, 1.20)	0.458
ACM including off-treatment data (With additional vital status follow-up)	98/4151 (2.36)	109/4134 (2.64)	0.89 (0.67, 1.16)	0.387
**Within 30 days**				
On-treatment ACM	0/4151 (0)	5/4134 (0.12)	NA	
ACM including off-treatment data	0/4151 (0)	5/4134 (0.12)	NA	
ACM including off-treatment data (With additional vital status follow-up)	0/4151 (0)	5/4134 (0.12)	NA	
**Within 60 days**				
On-treatment ACM	8/4151 (0.19)	6/4134 (0.15)	1.30 (0.45, 3.74)	0.628
ACM including off-treatment data	9/4151 (0.22)	8/4134 (0.19)	1.12 (0.43, 2.89)	0.822
ACM including off-treatment data (With additional vital status follow-up)	9/4151 (0.22)	8/4134 (0.19)	1.12 (0.43, 2.90)	0.818
**Within 180 days**				
On-treatment ACM	23/4151 (0.55)	19/4134 (0.46)	1.15 (0.63, 2.12)	0.647
ACM including off-treatment data	35/4151 (0.84)	34/4134 (0.82)	1.02 (0.63, 1.63)	0.946
ACM including off-treatment data (With additional vital status follow-up)	36/4151 (0.87)	35/4134 (0.85)	1.02 (0.64, 1.63)	0.931

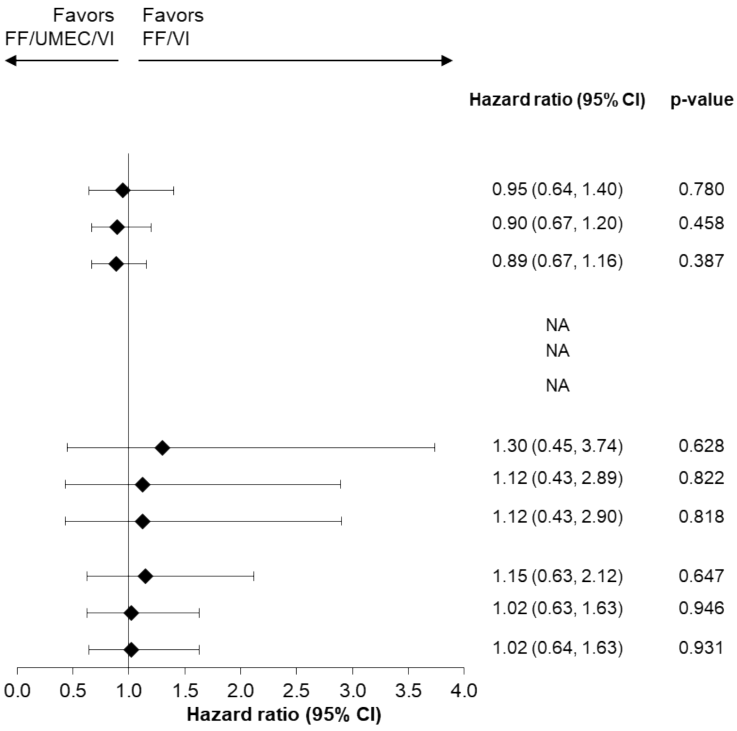

ACM, all-cause mortality; CI, confidence interval; FF, fluticasone furoate; UMEC, umeclidinium; VI, vilanterol. Note: Analysis was not performed if there were zero events in one or more of the three treatment arms (FF/UMEC/VI, FF/VI or UMEC/VI).
